# Vitamin D in the prevention, prediction and treatment of neurodegenerative and neuroinflammatory diseases

**DOI:** 10.1007/s13167-017-0120-8

**Published:** 2017-11-15

**Authors:** Priscilla Koduah, Friedemann Paul, Jan-Markus Dörr

**Affiliations:** 1 Charité – Universitätsmedizin, corporate member of Freie Universität Berlin, Humboldt- Universitäts zu Berlin, and Berlin Institute of Health, Neurocure Cluster of Excellence, Berlin, Germany; 2 Charité – Universitätsmedizin, corporate member of Freie Universität Berlin, Humboldt- Universität zu Berlin, and Berlin Institute of Health, Neurocure Cluster of Excellence and Experimental and Clinical Research Center, Max Delbrueck Center for Molecular Medicine and Charité – Universitätsmedizin Berlin, Berlin, Germany; 3 Charité – Universitätsmedizin, corporate member of Freie Universität Berlin, Humboldt- Universitäts zu Berlin, and Berlin Institute of Health, Neurocure Cluster of Excellence, and Multiple Sclerosis Center Hennigsdorf, Oberhavel Clinics, Berlin, Germany

**Keywords:** Vitamin D, Multiple sclerosis, Neuromyelitis optica spectrum disorders, Parkinson’s disease, Alzheimer’s disease, Predictive preventive personalized medicine

## Abstract

Vitamin D research has gained increased attention in recent times due to its roles beyond bone health and calcium homeostasis, such as immunomodulation. In some parts of the brain and on immune cells, vitamin D hydroxylating enzymes and its receptors are located. Epidemiological evidence demonstrates that deficiency of Vitamin D is relevant for disease risk and course in multiple sclerosis (MS) and presumably also in neuromyelitis optica spectrum disorders (NMOSD), Parkinson’s disease (PD), and Alzheimer’s disease (AD). Although the exact mechanism underlying vitamin D effects in these diseases remains widely unexplored, human and animal studies continue to provide some hints. While the majority of vitamin D researchers so far speculate that vitamin D may be involved in disease pathogenesis, others could not show any association although none have reported that sufficient vitamin D worsens disease progression. The studies presented in this review suggest that whether vitamin D may have beneficial effects in disease course or not, may be dependent on factors such as ethnicity, gender, diet, vitamin D receptor (VDR) polymorphisms and sunlight exposure. We here review the possible role of vitamin D in the pathogenesis and disease course of MS, NMOSD, PD, and AD and potential therapeutic effects of vitamin D supplementation which may be relevant for predictive, preventive, and personalized medicine. We suggest areas to consider in vitamin D research for future studies and recommend the need to supplement patients with low vitamin D levels below 30 ng/ml to at least reach sufficient levels.

## Introduction

Vitamin D has gained increased attention in diverse areas of biomedical research due to its role that goes beyond skeletal and calcium (Ca) metabolism [1, 2]. The impact of vitamin D has been studied in cardiovascular diseases [3], neuroinflammation [4, 5], and neurodegenerative diseases [6] among others. This is not surprising as the receptors of vitamin D and the enzyme (α1-hydroxylase) needed for its activation are located in many internal organs, immune cells, and also key areas of the brain [7].

Epidemiological evidence suggests that in many countries across the globe, vitamin D deficiency is prevalent across all age groups, irrespective of the geographical location [8] or seasonal changes [9] as well as in healthy subjects [10].

The endogenous metabolism of vitamin D is mediated by ultraviolet (UV) B radiation converting 7-dehydrocholesterol in the skin to cholecalciferol. Hydroxylation of cholecalciferol in the liver then leads to 25-hydroxyvitamin (25(OH)D with a subsequent hydroxylation step producing the active form 1,25-dihydroxyvitamin D3 also known as 1,25-dihydroxycholecalciferol (1,25(OH)2D) in the kidneys [11]. In this review, the use of the term vitamin D refers to 25(OH)D or 1,25(OH)2D3 unless otherwise stated.

Inadequate vitamin D levels are pertinent to the pathogenesis of multiple sclerosis (MS) and presumably also of neuromyelitis optica spectrum disorders (NMOSD), Parkinson’s disease (PD), and Alzheimer’s disease (AD). In all these diseases, it is reported that patients tend to have low serum vitamin D levels compared to healthy controls [12–14].

Prospective studies and meta-analyses have demonstrated that low serum or plasma vitamin D levels increased the risk of dementia [15, 16], cognitive impairment [12, 13], impaired motor functions [17, 18], and memory decline [19] which are all characteristics of neurodegenerative diseases. Additionally, evidence from cross-sectional studies has shown the impact of vitamin D deficiency on falls and balance in Parkinson’s disease (PD) [17].

In the context of autoimmune diseases, administration of vitamin D prevented the onset of experimental autoimmune encephalomyelitis (EAE), a rodent model of MS [20]. In an animal model of AD, dietary supplements with vitamin D enhanced learning and memory compared to healthy controls [21].

We here review the possible role of vitamin D in the pathogenesis and disease course of MS, NMOSD, PD, and AD and potential therapeutic effects of vitamin D supplementation which may be relevant for predictive and/or personalized medicine and may inform individualized treatment strategies for patients with these often debilitating neuroinflammatory and neurodegenerative diseases.

### Vitamin D beyond bone mineralization and Ca metabolism

Besides the fundamental effects of vitamin D on skeletal health and Ca homeostasis, several other important functions have been identified. In this section, we summarize the neurosteroid properties of vitamin D beyond Ca homeostasis in relation to neuroinflammatory, neurodegenerative, and autoimmune diseases.

The modulation of the innate and adaptive immune system is an important function of vitamin D [22–24]. Literally, all cells of the immune system express the vitamin D receptor as a prerequisite for being amenable to vitamin D signaling. Furthermore, many immune cells show 1α-hydroxylase activity, suggesting auto- and paracrine immune regulation via local 1,25(OH)2D3 vitamin D concentrations at the sites of inflammation. For further details, we refer the reader to a recent comprehensive review of the detailed effects of vitamin D on immune cell subsets [24]. Specifically, in MS an established important immunomodulatory effect of vitamin D is the induction of tolerogenic dendritic cells from human monocytes, which promote the differentiation of regulatory rather than pro-inflammatory T cells [25]. Additionally, it supports the differentiation of naïve CD4+ T cells into immunomodulatory T helper 2 (Th2) and regulatory T cells rather than pro-inflammatory Th1 and Th17 cells. It also interferes with antibody production by plasma cells [26] and inhibits the production of pro-inflammatory cytokines such as interferon gamma and tumor necrosis factor (TNF) alpha by cytotoxic CD8+ T cells.

Additionally, several lines of evidence point to the neuroprotective role of vitamin D. Increased activity of L-type voltage-sensitive calcium channels (LVCC) has long been shown to induce aging as well as increased cell death in the hippocampus of rats [27, 28]. Reports from animal studies and neuronal cell cultures have shown that 25(OH)D or the active metabolite 1,25(OH)2D3 prevents neurotoxicity by downregulating LVCC [29]. Specifically, in AD, vitamin D is shown to mediate the clearance of amyloid beta plaques (Aβ) through the activation of macrophages [30]. The clearance of Aβ protected against apoptosis which usually induces oxidative stress resulting in damage in the brain of AD patients [30, 31].

On the other hand, vitamin D is known to enhance neuronal survival by regulating neurotrophic factor-3 (NT-3) and Glial-derived neurotrophic factor (GDNF) synthesis in an animal model of AD [32, 33].

Not only is vitamin D presumably neuroprotective; it also plays important roles in synaptic plasticity. In an animal model of AD, administration of vitamin D restored the otherwise low extracellular postsynaptic potentials in the cornu ammonis 3, 1 (CA3, CA1) regions of the hippocampus [34] since vitamin D receptors are located here.

### Sunlight exposure and disease risk

Several studies have found that sunlight exposure attenuates disease course in many diseases such as MS and PD among others due to its influence on circulating vitamin D levels. UV radiation accounts for 90% of serum vitamin D while the contribution of dietary sources is less relevant [35, 36]. In PD, exposure to sunlight was shown to enhance bone mineral density by boosting vitamin D levels in the serum [37]. A population-based study also showed an increase in disability worsening in MS patients with reduced sunlight exposure [38].

The positive correlation between MS prevalence in different areas of the world and latitude is long known [39]. Interestingly, a link between sun exposure and MS incidence can be observed already on a less global level. In France for example, MS prevalence shows a strong inverse association with the mean annual UVB radiation as southern areas with higher annual UVB radiation levels show lower MS prevalence than northern areas with lower UVB radiation levels [40]. In regions of higher latitude (50.0–56.0°), the onset of MS was found to be 2 years earlier than those in lower latitudes (19.0–39.9°) [41]. Exception from the latitude gradient rule can be at least partially explained by genetic differences and/or lifestyle including dietary variations [42]. For example, a lower MS prevalence than one would expect from the latitude was observed in regions such as Norway with a high consumption of fatty vitamin D-rich fish [43]. A recent study supplemented relapsing-remitting MS patients with deficient levels of serum vitamin D (≤ 24 nmol/l or ≤ 9.6 ng/ml). It was found that each nanomoles per liter increase in serum vitamin D levels lead to a − 0.014 (95% CI − 0.026 to − 0.003) reduction in the annualized relapse rate [44].

Several studies including a recent meta-analysis demonstrated that spring borns have a significantly higher lifetime MS risk than those born in autumn, which has been attributed at least in part to an insufficient in utero vitamin D supply because of low maternal vitamin D serum levels during winter [45–48]. Likewise, the level of sun exposure during childhood and adolescence, e.g., by outdoor leisure activities which may serve as a proxy for vitamin D supply in early life, has been inversely linked to the risk of MS in adulthood [49–52]. Although tempting, these studies are limited by the inevitably biased retrospective determination of past sun exposure and the possibility of intrinsic vitamin D independent effects of sun or UVB exposure itself on the disease [53–55].

In the next sections, we review the role of vitamin D in MS, NMOSD, PD, and AD.

## The role of vitamin D in multiple sclerosis and EAE

MS is considered the most common chronic inflammatory disorder of the CNS in western countries and — as it is an incurable disease — a leading course of disability and early retirement in young adults, predominantly females of child-bearing age [56–59]. Worldwide, about 2.5 million people suffer from MS [60]. Clinically, MS is characterized by recurrent subacute bouts of CNS dysfunction including but not limited to central motor system dysfunction, optic neuritis, coordination, balance and sensory disturbances, cognitive dysfunction [61], and fatigue [62–64]. Pathophysiologically, autoreactive encephalitogenic T cells in concert with B cells and cells of the innate immune system orchestrate an autoimmune reaction against CNS structures which results in demyelination and neuroaxonal damage of the brain, retina, and spinal cord from earliest disease stages on [65–72]. Both genetic and environmental factors contribute to the individual risk of MS. Several lines of evidence which are discussed in more detail below suggest vitamin D as an environmental factor to impact both the risk to develop MS as well as the course of the already established disease. In experimental autoimmune encephalomyelitis (EAE), the animal model of MS, vitamin D was shown to modulate both innate and adaptive immune responses and has prophylactic and therapeutic effects.

In the next section, we discuss the (1) geographical and seasonal associations of vitamin D with the risk and course of MS, (2) associations between vitamin D supply or serum levels in early years and the risk to develop MS later, (3) relations between serum levels and clinical and/or radiographical disease activity, and finally, (4) data from interventional clinical trials.

### Prophylactic and therapeutic effects of vitamin D in EAE

In the active EAE model which is still the best established rodent animal model for MS [73], immunization of healthy mice with myelin proteins results in MS-like CNS inflammation and ascending flaccid paresis after several days. Dietary treatment of mice with vitamin D starting from disease induction (prophylactic paradigm) resulted in a reduced incidence and severity of the disease. Likewise, starting vitamin D treatment at the onset of symptoms (therapeutic paradigm) ameliorated the course and severity of the disease. Withdrawal of vitamin D leads to a resumption of disease activity [20, 74]. These early studies suggested that higher serum vitamin D levels are both preventive and therapeutic in autoimmune encephalomyelitis. Interestingly, in a later study, benefits of vitamin D were only observed in female but not in male mice which suggest gender differences in the protective role of vitamin D at least in the animal model [75]. Clearly, these results cannot be directly transferred to the human situation.

### Relations between vitamin D levels and disease activity

Does vitamin D level impact the risk of MS, and if so, what is the most vulnerable period during a lifetime? These important questions are methodologically difficult to answer. Prospective population-based studies do not exist, and epidemiological or observational studies are usually limited by retrospective design, selection bias, and interference with various confounders. A nested case-control study using stored serum samples from US Army members suggested a decreasing risk of MS with increasing past vitamin D serum levels. In fact, a 50 nmol/l (20 ng/ml) increase in serum 25(OH)D decreased the odds to develop MS to 0.59 (95% confidence interval, CI 0.36–0.97) in the white population. In black and Hispanic populations, there was no significant association between vitamin D levels and MS risk with odds of 0.66 (95% CI 0.24–1.78; *p* = 0.41) and 0.97 (95% CI 0.28–3.33; *p* = 0.96), respectively [76]. Likewise, in a large-scale long-term cohort study, the risk of MS was inversely associated with the dietary vitamin D intake. The relative risk in the highest quintile of total vitamin D intake compared with those in the lowest was 0.67 (95% CI 0.40 to 1.12) [77]. Extending the above discussed data on childhood and adolescence as critical periods, recent data suggest that already in the uterus supply of vitamin D and, as recently shown in a Danish population-based case-control study, neonatal vitamin D may impact the risk of MS later in life [78, 79].

In established disease, several lines of evidence consistently suggest that higher serum levels are associated with a more favorable disease course, i.e., fewer relapses and less MRI activity. MS patients have lower serum levels than matching healthy controls, and lower levels can be detected during relapses compared to phases of remission [80]. Importantly, levels are already lower at disease onset, which argues against insufficient levels being a consequence of disease (reverse causality), for example by reduced sunshine exposure due to disability with subsequently reduced mobility (less outdoor activities) [81]. Several studies consistently showed an inverse relationship between vitamin D levels and frequency of relapses, disability progression, and occurrence of new brain MRI lesions [82–88]. In one study, for example, doubling of serum levels were associated with a reduction of relapse rate by 27% [82], and in another study in pediatric MS, every 10 ng/ml increase in vitamin D levels was associated with a 34% reduction of relapse rate [84]. Furthermore, in patients with a first demyelinating event suggestive of MS, a 50 nmol/l increment of average serum levels within the first 12 months predicted significantly lower rates of relapses, new active lesions, and a trend of lower brain volume loss in the following 5 years [87]. A very interesting recent study suggested a relation of the severity of neuro-axonal retinal damage after optic neuritis and serum vitamin D levels, in that retinal damage was substantially lower in patients with high vitamin D levels with men having greater retinal nerve fiber layer and ganglion cell layer thinning than women [89]. In summary, data consistently show a clear association between higher vitamin D levels with a less active disease course. However, data are not sufficient to prove a causal relationship.

### Interventional clinical trials

The few interventional trials undertaken so far to investigate whether vitamin D supplementation can beneficially influence the disease course have been methodologically fairly heterogeneous, and study designs have not always been appropriate. Some studies were not longitudinal, and other confounding factors such as the outdoor activity of patients and sunlight exposure were not considered. Not surprisingly, results of these trials are rather inconsistent. A small and uncontrolled study focusing on safety demonstrated that short-term doses up to 280,000 IU/week over a 28-week period are safe in MS. A possible effect on gadolinium-enhancing MRI lesions but not on clinical outcome parameters was observed [90]. Two randomized double-blind and placebo-controlled short-term trials showed a significant shift from a pro- to a rather anti-inflammatory serological cytokine pattern [91, 92]. Another randomized and controlled but open-label study comparing doses up to 40,000 or 4000 IU cholecalciferol per day for 12 months in 49 MS patients showed a significantly reduced relapse rate in the high dose arm. Two randomized, double-blind and placebo-controlled studies using 20,000 IU cholecalciferol per week also failed to detect significant effects on clinical parameters of disease activity although one study showed significantly fewer gadolinium-enhancing lesions on brain MRI [93, 94]. Another study investigated vitamin D2 (VD2) effect in relapsing-remitting MS patients. All participants received 1000 IU VD2 daily (on ethical grounds to prevent deficiency) while the high-dose arm additionally received 6000 IU for a period of 6 months. The results from this study could not show any effect of vitamin D2 in terms of clinical and MRI parameters [95]. Accordingly, a meta-analysis of five clinical trials found no significant effect of high-dose vitamin D on the risk of relapse in MS [96]. Results of the randomized double-blind and placebo-controlled SOLAR (NCT01285401) study, which addressed the effect of 14,000 IE cholecalciferol add-on to interferon beta on clinical and MRI parameters in 229 RRMS patients, were presented at the 2016 ECTRIMS meeting in London, UK. While the primary endpoint (no evidence of disease activity) was missed, a trend to a 30% relapse rate reduction, not reaching statistical significance, and a significant 32% reduction of active MRI lesions (both gadolinium-enhancing and T2 lesions) was reported.

A US single-center randomized double-blind control trial involving 40 MS patients between 18 and 55 years, receiving either a high dose (10,400 IU) or low dose (800 IU) daily for 6 months, showed reduced IL-17 production and effector memory CD4+ T cells in the higher dose arm. This study, although with small sample size, adds to the knowledge on the immunomodulatory effect of vitamin D in MS [97]. However, the question whether vitamin D positively impacts the course of MS is still not answered. Several interventional studies investigating the effect of vitamin D on disease severity either by supplementation with vitamin D as add-on therapy or UVB phototherapy are ongoing [98–101].

The EVIDIMS study is a German multi-center randomized double-blind phase II trial which aims to investigate the effect of vitamin D supplementation as add-on therapy to interferon-β1b treatment with patients grouped into high-dose and low-dose arms [99] while the VIDAMS trial investigates the effect of vitamin D on MS after a 1 month treatment with glatiramer acetate in high-(5000 IU) and low-dose arms (600 IU) [98].

In conclusion, meanwhile, a huge body of evidence points towards a less active course of MS in the presence of high serum vitamin D. However, the ultimate question of whether and how much vitamin D MS patients should take can still not be answered on scientific grounds. Additionally, a “real-world” study investigating the association between serum vitamin D levels on disease course in patients with disease-modifying drugs of MS such as interferon-β, glatiramer acetate and fingolimod found differences in the effect of serum vitamin D levels on the relapse rate and gadolinium-enhancing lesions in the different treatment classes. The risk of relapse was lower in the interferon-β cohort alone (HR, 0.58) or in the combined interferon-β and glatiramer acetate cohort (HR = 0.77) compared to the glatiramer acetate cohort alone (HR = 0.89). Also, the effect of vitamin D on gadolinium-enhancing lesions was more pronounced in the interferon-β treated group (HR = 0.41). In fingolimod treatment patients, serum vitamin D did not have any significant effect on gadolinium-enhancing lesions. This suggests that the effect of vitamin D may also be influenced by the type of therapy the patient is already receiving [102]. In a randomized double-blind controlled trial, the effect of alfacalcidol, a synthetic analogue of vitamin D on MS-related fatigue (assessed by the Fatigue Severity Scale, FSS), was investigated in patients with mean age 41.1 ± 9.2 years and disease duration of 6.2 ± 5.5 years. Treatment with 1 mcg/d of alfacalcidol for 6 months reduced the mean relative fatigue score to − 41.6% compared to − 27.4% in the placebo group with improved quality of life [103]. In sum, patients with inadequate vitamin D levels (< 30 ng/ml / 75 nmol/l) should either be supplemented with vitamin D or should be exposed to more sunlight in order to achieve levels between 100 nmol/l (40 ng/ml) and 150 nmol/l (60 ng/ml) [4, 104, 105].

## Neuromyelitis optica spectrum disorders

Neuromyelitis optica spectrum disorders (NMOSD) are inflammatory autoimmune conditions of the central nervous system predominantly affecting the optic nerve, brainstem, and spinal cord; however, fatigue, pain, depression, and sleep problems are also frequent symptoms [106–109]. For a long time, NMOSD were considered rare variants of MS; however, the seminal detection of a highly specific serum biomarker, antibodies to the astrocyte water channel aquaporin-4, in up to 80% of patients with a NMOSD phenotype, as well as subsequent immunological, biomarker, and imaging studies made clear that NMOSD is an autoimmune disease entity distinct from MS [110–117]. Accrual of irreversible neurological disability is usually faster and long-term prognosis poorer than in classical MS [118, 119]. For clinical management, it is important to bear in mind that many classical immunomodulatory drugs given in MS are inefficacious or even harmful in NMO [120–123]. Recently, antibodies to myelin oligodendrocyte glycoprotein (MOG) were reported in a subset of patients with a NMOSD phenotype seronegative for aquaporin-4 antibodies [124–127] and some patients with MS [128, 129]. There is an ongoing scientific debate as to whether adult patients with MOG antibodies should be diagnosed with NMOSD or whether MOG-antibody associated encephalomyelitis is a disease entity in its own [130, 131].

Few studies have investigated vitamin D levels in patients with NMOSD. One study from Korea reported significantly lower vitamin D levels in 51 NMOSD patients seropositive for aquaporin-4 antibodies as compared to 204 healthy controls [132]. Although in patients, there was no difference in vitamin D levels between relapse and remission and no association with the annualized relapse rate, higher vitamin D levels were associated with less neurological disability measured by the Expanded Disability Status Scale (EDSS). A recent study from Thailand compared vitamin D levels in 20 patients with clinically isolated syndrome (first manifestation of MS), 34 patients with MS, and 76 patients with NMOSD [133]. Prevalence of vitamin D insufficiency and deficiency (< 30 ng/ml or 75 nmol/l) ranged from 73 to 80%, without a difference between groups and no association with disease activity and neurological disability. Conversely, a Chinese study reported lower vitamin D levels in 58 NMOSD patients versus 116 healthy controls and lower levels in 43 NMOSD patients measured during relapse compared to 15 NMOSD patients in remission [134]. The authors moreover reported an inverse correlation between the EDSS during attacks and vitamin D levels. Another study from Turkey found lower vitamin D levels in 24 NMOSD patients and 19 MS patients compared to 22 healthy controls [135]. Neither did aquaporin 4 antibody status influence vitamin D levels nor was there a correlation between vitamin D levels and age, number of relapses, and EDSS scores. It is important to keep in mind that none of these studies was longitudinal in design, and it is thus not possible to adequately address the problem of reverse causation discussed in the MS section above. Two earlier studies had investigated the association between vitamin D levels and inflammatory spinal cord disease, a subgroup of which had a final diagnosis of NMOSD [136, 137]. Vitamin D levels were significantly lower in patients who had monophasic spinal cord inflammation compared to recurrent disease, and vitamin D insufficiency (< 30 ng/ml) was associated with a fourfold (CI 1.60–10.0) increased risk of recurrence, along with other predictors of recurrent disease. To date, there are no studies that specifically investigated vitamin D levels in patients with MOG antibodies.

In sum, despite inconclusive results from the few studies above, it is in light of the devastating disease course of many NMOSD patients and the significantly increased risk of recurrence following a first episode of myelitis in case of vitamin D insufficiency advisable to measure vitamin D levels in patients with NMOSD and to raise these to above 30 ng/ml (75 nmol/l).

## Role of vitamin D in Parkinson’s disease

Parkinson’s disease (PD) is a neurodegenerative disease of the central nervous system characterized by tremor, rigidity, bradykinesia, postural imbalance resulting in falls, and severe psychiatric symptoms like depression and dementia [138–140]. It is the second most common progressive neurodegenerative disease affecting more men than women with the incidence rate differing between ethnicities and increasing with age [141]. Although the exact causes remain elusive, genetic and environmental factors contribute to the disease risk. PD patients tend to have lower vitamin D levels associated with low bone mineral density contributing to disease-related disability [37].

Recent epidemiological studies have shown that low vitamin D levels are more prevalent in PD patients compared to healthy subjects, and low serum or plasma vitamin D may be a predictive marker for PD risk and severity. In PD patients with a mean age of 65 years, a 400 IU per day vitamin D supplementation should reduce the risk of fractures by 20% [142]. Studies from North America and China investigating the role of vitamin D levels in PD patients found an association between plasma and serum vitamin D levels and disease severity (measured by the Hoehn and Yahr stage and the Unified Parkinson’s Disease Rating Scale (UPDRS)). In the North American study, 17.6% of the 388 PD patients had vitamin D deficiency (< 30 ng/ml (75 nmol/l) [10] compared to 9.3% of the 283 age-matched healthy controls (30.6 ng/ml) [143]. In this study, not all patients had vitamin D deficiency and some healthy controls were also not vitamin D deficient. This draws attention again to the issue of reverse-causation as explained above. The studies failed to assess the activities of patients and even healthy controls with regard to sunlight exposure, outdoor activities, or even dietary vitamin D intake.

In the Chinese cohort involving 229 PD patients and 120 age-matched healthy controls, serum vitamin D levels were significantly lower (< 20.6 ng/ml) in PD patients compared to 22.9 ng/ml in healthy subjects [144].

### Therapeutic effects of vitamin D in animal models of PD

The loss of dopaminergic neurons is a major characteristic of PD; hence, therapeutic approaches have focused on finding ways to restore the dopaminergic neurons in the substantia nigra [145].

Animal models of PD have been often used to test the efficacy of vitamin D in the disease pathology in terms of dopamine production, neurotrophic factors synthesis, as well as the possible neuroprotective effect of vitamin D. In a rat model of PD, intraperitoneal injection of 1,25(OH)2D3 before or after inducing PD partially restored tyrosine hydroxylase expression in the substantia nigra [146] thus promoting the conversion of tyrosine to dopamine. Another rodent study, investigating the effect of vitamin D on zinc-induced neurotoxicity in the substantia nigra of rats, found that vitamin D reduced oxidative injury in the substantia nigra [147].

Genetic studies focusing on vitamin D receptor polymorphisms have also shown an association between vitamin D and PD [148–150]. The response to vitamin D treatment may be partially or fully influenced by VDR gene variants. Using healthy mice to test the effect of vitamin D on motor functions, vitamin D receptor knockout mice showed impaired motor functions evident in the swim test, with significantly reduced immobility duration compared to the wildtype counterparts [151].

### Interventional and genetic clinical studies

There are very few interventional studies that have been undertaken to investigate a potentially beneficial effect of vitamin D on the disease course in PD. The human studies have focused on the polymorphisms of vitamin D receptors. The findings from most human observational studies have been inconsistent due to different population characteristics and confounding variables such as seasonal changes, diet, and genetic variations of the VDR among others. While most studies have shown that sufficient vitamin D status (> 30 ng/ml) and the expression of particular VDR gene variants may ameliorate disease severity, one study conducted in the Faroe Islands could not establish any relationship between serum 25(OH)D or VDR polymorphisms on PD [152].

In a 29-year longitudinal study involving 3173 men and women between the ages of 50 to 79 years from Finland who were free from PD at baseline, individuals with high serum vitamin D levels (> 20 ng/ml or at least 50 nmol/l) had 65% reduced risk of PD compared to those with low serum vitamin D levels (< 8 ng/ml or 25 nmol/l) [6]. Moreover, some of the clinical presentations of PD such as falls, fractures, and balance problems have been attributed to low levels of circulating vitamin D [17, 148, 153].

The link between vitamin D and PD worsening could be explained by the fact that VDRs are highly expressed in the substantia nigra [7], a key anatomical region involved in PD pathogenesis.

The VDR which is located on chromosome 12q13.1 has numerous polymorphisms located on different exons at the promoter region of the VDR. The most characterized are the FokI allele with different variants such as the homozygous FokI TT/CC or a heterozygous FokT/C on exon 2, the BsmI on exon 8, and the ApaI and TaqI on exon 9 [154, 155].

Different alleles of the VDR have been shown to affect PD differently. Suzuki and colleagues in a randomized double-blind study involving 104 PD patients who received 1200 IU of vitamin D per day for 12 months assessed the effect of vitamin D in PD [148]. In this study, the Hoehn and Yahr scale and the UPDRS were used to assess disease severity. Patients that received vitamin D supplements had better disease rating compared to the placebo group. Investigating the effect of vitamin D on the genetic level revealed that carriers of the FokI TT allele responded better to vitamin D treatment, the FokI CT carriers had moderate effect, while the FokI CC carriers [148] showed no effect of vitamin D.

In contrast, two other studies investigating the effect of VDR polymorphisms in PD found that the FokI T/C allele was associated with PD. The FokI C allele was predominant in PD patients than in healthy controls in Chinese and Hungarian populations [149, 150].

In conclusion, evidence from both human and animal studies point to a beneficial effect of vitamin D in PD, although few studies suggest otherwise.

## Role of vitamin D in age-related cognitive decline and Alzheimer’s disease

Alzheimer’s disease is a neurodegenerative disorder affecting mostly elderly persons resulting in cognitive decline and dementia [156, 157]. Worldwide, about 24 million people suffer from the disease, and this number is expected to double by 2040 [158].The neuropathology of the disease is characterized by neurofibrillary tangles and senile plaques in the brain. A major hallmark of the disease is the accumulation of Aβ plaques, a component of senile plaques caused by the proteolysis of amyloid-beta protein precursors [159]. It is speculated that the accumulation of Aβ induces inflammatory responses which account for the neurodegenerative component of the disease resulting in cognitive decline [160, 161]. Although the causes of AD remain unknown, environmental and genetic factors have been suggested to be associated with the disease [162].

The most characterized genetic cause is attributed especially to the epsilon 4 (ε4) allele of apolipoprotein E (APOE) [163, 164]. However, not all AD patients express the APOEε4 allele which points to additional causative factors [165, 166]. For example, low serum vitamin D levels (< 30 ng/ml) and polymorphisms of the vitamin D receptor have also been associated with AD.

### Genetic studies of vitamin D in AD

The effect of vitamin D in AD can be explained by the fact that VDRs are located in several regions of the brain, and low VDR mRNAs were found in the brains of AD patients postmortem [167].

Most of the studies that have investigated the role of vitamin D in Alzheimer’s disease were observational or genetic studies.

A genetic study by Gezen and colleagues involving 104 late-onset AD patients showed that single nucleotide polymorphisms (SNPs) in VDR altered the vitamin D- receptor pathway. The ApaI but not the TaqI polymorphism of the VDR was found to be associated with AD [168, 169]. Other studies focusing on the genetic variants of the VDR found associations between the BamI, TaqI alleles, and cognitive decline, while the ApaI variant was associated with better cognition [170].

Following these findings, Lehmann and colleagues found that the ApaI and TaqI alleles were associated with risk of AD with odd ratios of 3.1 (95% CI 1.4–6.7, *p* = 0.005) and 3.0 (95% CI 1.6–5.6, *p* = 0.0008) for patients below 75 years, respectively [171]. These findings were confirmed in a study involving Polish and British populations where carriers of TaqI, ApaI, FokI, and BsmI showed a lower risk of AD [172]. However, these alleles, especially ApaI, were not associated with AD in the Turkish and Iranian population suggesting that the association of VDR polymorphisms with AD may be influenced by the ethnicity of the patient [172].

### Interventional studies in animal models of AD

Interventional animal studies where rats received vitamin D as dietary supplements (10,000 IU/Kg/day) for 5 to 6 months showed that rats with vitamin D supplement performed better in the Morris water maze test, a hippocampal-dependent learning and memory task, compared to controls [21]. On the other hand, cognitive impairment worsened when vitamin D was removed from the diet of animals [21]. Furthermore, subcutaneous injections of 1,25(OH)2D3 reduced age-related cognitive impairment in rats and also increased clearance of Aβ plaques in older rats compared to the younger ones [173].

The exact mechanism by which vitamin D mediates AD is not clearly established. However, vitamin D reduces oxidative stress thus preventing neurons from dying through the activation of macrophages which help Aβ plaques clearance as shown in in vitro studies [30, 31]. One of the basic functions of vitamin D is Ca homeostasis. However, in AD, the presence of Aβ activates low-voltage calcium channels (LVCC) leading to Ca imbalance. In animal studies, vitamin D enhanced Ca homeostasis and protected neuronal death by downregulating LVCC induced by Aβ [34]. Offspring of rats depleted of vitamin D supplementation had reduced nerve growth factor and glial-derived neurotrophic factor, and this was linked with abnormal brain morphology such as enlarged ventricles [174]. The clinical consequence is that pregnant women should have adequate vitamin D levels to avoid any developmental defects.

### Interventional clinical studies

A study in the USA investigated 1658 elderly adults (mean age of 73.6 years) free from dementia or any cardiovascular disease followed over a mean of 5.6 years (SD 1.6, median 6.1, range 0.1–8.4). Participants with severe vitamin D deficiency (vitamin D levels < 25 nmol/l or 10 ng/ml) had higher hazard ratio of 2.25 (95% CI 1.23–4.13), while participants with vitamin D levels above 25 nmol/l–50 nmol/l (10 ng/ml–20 ng/ml) had hazard ratios of 1.53 (95% CI 1.06–2.21) compared to those with sufficient vitamin D levels (≥ 50 nmol/l or ≥ 20 ng/ml) to develop all-cause dementia. Of the 171 participants who developed all-cause dementia, there were 102 cases of AD [15].

A randomized controlled double-blind placebo trial investigated the effect of vitamin D on cognition in healthy elderly subjects above age 65 years. The vitamin D-supplemented group (supplement contained 4.0 μg (160 IU) vitamin D and other trace elements) had better cognitive performance (*p* < 0.01) in the different cognitive tests (7 cognitive tests including the Mini-Mental State examination) compared to the placebo group [175].

A French study investigating the effect of dietary vitamin D intake on cognition in older women with mean age of 75 years and followed for 7 years showed that higher dietary intake of vitamin D of 2336.41 IU weekly (mean baseline dietary intake of 58.41 ± 30.09 μg/week (range 2.53–205.54)) reduced risk of AD with adjusted odd ratios of 0.23 (95% CI 0.08–0.67) compared to those with lower baseline vitamin D intake [176]. A limitation of this study is the fact that self-administering of dietary vitamin D may result in false reports or perhaps insufficient quantities of vitamin D intake which may introduce variations within the same group.

Furthermore, a meta-analysis of 37 studies comparing the association between vitamin D levels and cognition using the Mini-mental State Examinations scores showed a weak association between higher vitamin D levels (≥ 50 nmol/l or ≥ 20 ng/ml) with hazard ratio of 1.2 (95% CI 0.5 to 1.9: *I* ^2^ = 0.65; *p* = 0.002) and cognitive performance. The authors explained that the heterogeneity in the methods used in assessing vitamin D concentrations may account for this [12].

In contrast to these findings, a randomized controlled trial investigating the effect of vitamin D on cognitive and emotional functioning in healthy young adults of mean age 21.8 years who received 5000 IU of vitamin D daily for 6 weeks could not show any positive association between vitamin D and cognition [177]. The study is, however, limited by the fact that participants were free from any chronic neurological disease and had sufficient serum vitamin D levels (> 76.6 nmol/l or > 30 ng/ml). Conversely, most studies assessing the effect of vitamin D on cognition have focused on the elderly populations with chronic neurological diseases and concomitant low vitamin D levels.

However, this throws more emphasis on whether vitamin D levels decrease with age and as to whether prolonged low levels of vitamin D are associated with the incidence of diseases in the adulthood thus highlighting the need to investigate the effect of reverse causation more thoroughly.

Nevertheless, patients with cognitive impairments should be tested, and those with low levels (< 30 ng/ml) should be supplemented.

In summary, vitamin D supplementation should be encouraged in the elderly as well as AD patients to combat the possible detrimental sequelae of low serum vitamin D levels.

## Conclusion and expert recommendations

Vitamin D deficiency presumably plays a causative role in the pathogenesis and course of various neuroinflammatory and neurodegenerative diseases. However, details of vitamin D involvement particularly on the individual level remain to be clarified, and evidence for vitamin D supplementation in indications other than bone health are confined to a few interventional clinical trials conducted so far. Additionally, results of these trials are at least in part inconclusive. As a result, for the time being, evidence-based recommendations on vitamin D supplementation doses or target serum levels are hardly possible. Serum vitamin D levels of 75 nmol/l (30 ng/ml) are safe and may potentially exert therapeutic effects in some patients with neurodegenerative and neuroinflammatory diseases such as reduced disability worsening, improved bone health, and improved cognition. Whether higher serum levels and/or a personalized high-dose vitamin D supplementation are superior in the prevention or modification of individual disease courses remains to be clarified. Additionally, prospective studies are needed to evaluate the eligibility of vitamin D-related parameters in models or tools to predict the development or course of diseases on an individual level.

Taking a more conservative point of view, we, therefore, recommend for the time being that patients are tested for vitamin D levels and that those with low levels are supplemented in order to reach serum levels of about 75 nmol/l (30 ng/ml). This target level may potentially be raised when future studies suggest higher levels to be beneficial.
